# Interventions for sleep difficulties in adults with an intellectual disability: a systematic review

**DOI:** 10.1111/jir.12587

**Published:** 2019-01-09

**Authors:** P. J. Shanahan, S. Palod, K. J. Smith, C. Fife‐Schaw, N. Mirza

**Affiliations:** ^1^ Department of Psychological Sciences, School of Psychology, Faculty of Health and Medicine University of Surrey Guildford UK; ^2^ Neurodevelopmental Services Your Healthcare CIC Surbiton UK; ^3^ Sutton and Merton Mental Health Intellectual Disability Team Jubilee Health Centre Wallington UK

**Keywords:** adult, insomnia, intellectual disability, sleep, treatment

## Abstract

**Background:**

Current literature highlights higher prevalence rates of sleep difficulties amongst adults with an intellectual disability. However, no synthesis has been conducted to assess the effectiveness of existing interventions in this population. Thus, the aim of this review was to assess the effectiveness of sleep interventions in adults with an intellectual disability (ID).

**Method:**

Eight databases were searched to identify interventions for sleep difficulties amongst adults with an ID. The study quality was assessed with the Risk Of Bias In Non‐randomised Studies – of Interventions. Nine studies (*n* = 97) were eligible for inclusion in the review.

**Results:**

There was a notable study on heterogeneity in terms of the population, study design, intervention studied, sleep assessment and outcome assessments used. Eight of the nine studies reported improvement in sleep following intervention. However, these findings need additional support as only 97 participants involving a variety of interventions and measurement systems were used across all studies. Furthermore, eight of the nine studies had serious to critical risk of bias. The only study identified as having low risk of bias was a placebo‐controlled randomised controlled trial for the use of melatonin.

**Conclusions:**

This review highlights the need for objective measures such as actigraphy and studies with greater experimental control investigating sleep interventions in adults with ID.

## Background

Sleep difficulties affect the amount, quality and timing of sleep (World Health Organisation 1992) and can affect up to 30% of the general population (Ferrie, Kumari, Salo, Singh‐Manoux & Kivimäki 2011). The implementation of evidence‐based sleep intervention strategies can help to reduce some of the short‐term and long‐term health and behavioural consequences of poor sleep (Cappuccio, D'Elia, Strazzullo & Miller 2010; Cappuccio, Cooper, Delia, Strazzullo & Miller 2011; Irwin, Olmstead & Carroll 2016). However, intervention strategies that are advocated for in the general population (e.g. cognitive behavioural therapy) may not always be appropriate or accessible for specialised populations such as adults with an intellectual disability (ID).

A systematic review of the prevalence of sleep difficulties amongst adults with ID identified that 32% experienced multiple sleep difficulties (Boyle *et al*. 2010 *;* Van de Wouw, Evenhuis & Echteld 2012). Using the Diagnostic and Statistical Manual of Mental Disorders Fourth Edition diagnostic criteria for insomnia, the prevalence was found to increase with age (Phillips & Mannino 2005). Previous work also indicates that adults with an ID have significantly less stable and more fragmented sleep than adults from the general population (Maaskant, van de Wouw, van Wijck, Evenhuis & Echteld 2013). Furthermore, evidence indicates that individuals with higher severity or co‐morbid disabilities are at an increased likelihood of having sleep difficulties (Lindblom *et al*. 2001).

In addition to the broad health consequences of sleep difficulties, there is evidence that adults with an ID can also exhibit challenging behaviours as a result of issues with their sleep (Lenjavi, Ahuja, Touchette & Sandman 2010). In particular, adults with higher support and communication needs present with an increased frequency of behaviours that challenge as a result of difficulties with their sleep (Vollmer & Smith 1996; Matson, González, Terlonge, Thorson & Laud 2007; Lenjavi *et al*. 2010). These challenging behaviours are associated with a higher cost of support and higher utilisation rates of day centres and specialists in community teams (Knapp, Comas‐herrera, Beecham & Pendaries 2005; Griffith & Hastings 2014).

Despite evidence supporting the prevalence and issues associated with sleep difficulties in adults with an ID, there is a need for more clarity around the evidence that supports existing recommendations. Within the UK, the National Institute for Health and Care Excellence (2015) recommends the use of functional analysis of problem sleep behaviours and structured bedtime routines prior to medication such as melatonin. However, the evidence base underpinning the use of this approach has primarily focused on studies involving children (O'Reilly 1995; Hanley, Iwata & McCord 2003). Within the UK, the use of melatonin for adults with an ID is currently licenced for those aged 55 or over and for a maximum of 13 weeks (National Institute for Health and Care Excellence 2015); thus, it is a commonly used intervention for insomnia.

Interventions typically utilised following assessment involve sleep hygiene and stimulus control. Sleep hygiene focuses on lifestyle and environmental factors such as reducing the consumption of caffeine, exercising regularly, avoiding daytime naps and managing stress to promote sleep (Irish, Kline, Gunn, Buysse & Hall 2015). Stimulus control reduces stimuli that promote wakefulness in the sleeping environment. Examples include avoiding the use of a television in the sleep environment and leaving the sleeping environment after 15 min if unable to sleep until feeling sleepy (Gunning & Espie 2003). Other interventions that may be effective in the adult population include optimal scheduling of sleep, light therapy and a multicomponent behaviour plan based on the function of sleep disrupting behaviour (Van de Wouw *et al*. 2012).

Existing syntheses on the effectiveness of sleep interventions in ID combine results from adult and child populations (Van de Wouw *et al*. 2012; Priday, Byrne & Totsika 2017). Therefore, there is a need for research that systematically examines the effectiveness of sleep interventions for adults with an ID. The aim of this work was to systematically determine the effectiveness of existing sleep interventions for adults aged 18+ with an ID.

## Methods

### Search strategy

A systematic literature search was performed in June 2017 using search terms categorised into ‘intellectual disability’, ‘sleep’ and ‘adult’ (Table 1). Search terms were combined using the Boolean operators ‘and’ between categories and ‘or’ within categories. Only those papers published between January 1997 and August 2017 were considered in the first search. The 20‐year time frame was chosen based on the American Academy of Sleep Medicine's conclusion that actigraphy was a useful research tool for the study of sleep in 1995 (Martin & Hakim 2011). Only papers published in English were reviewed. The search terms were entered into the following electronic databases: BNI (Proquest, Michigan, USA), CINAHL (Elsevier, Amsterdam, Netherland), HBE (EBSCO, Ipswich, USA), HMIC (OVID, Leeds, UK), MEDLINE (EBSCO, Ipswich, USA; and Proquest, Michigan, USA), PsychArticles (American Psychological Association, Washington DC, USA), PsychINFO (American Psychological Association, Washington DC, USA) and PubMed (United States National Library of Medicine, Bethesda, MD, USA). A second search was completed using the criteria published in June 2017 in specialised journals including *Advances in Psychiatric Treatment*, *The Psychiatrist* and *British Journal of Psychiatry* (see Table 1 for a list of all the search terms used). A third search was completed to review papers published between June 2017 and August 2018. References of the included studies were also reviewed.

**Table 1:** Search strategy (Boolean operators ‘or’ and ‘and’ between columns)

Intellectual disability	Sleep	Adult (>18 years)
intellectual disability, intellectually retarded, intellectually disabled, mental disability, mentally disabled, idiocy, mental deficiency, learning disability, learning disorder, learning disturbance, developmental disability, mental handicap, mentally handicap, intellectual handicap, intellectually handicap, Down syndrome, mental incapacity, intellectual incapacity and oligophrenia	sleep, sleep disorder, insomnia, dyssomnia, parasomnia, parasomnias, somnolence, hypersomnia, circadian, wake, ultradian, night terrors, sleepwalking, somnambulism, nightmares, sleep apnoea, nocturnal, hypnotics, soporific, REM, nap, narcolepsy, snoring and sleep paralysis	adult, middle aged, ageing, elderly, geriatric, old and senior

The protocol is available on PROSPERO with ID: CRD42017076262 (https://www.crd.york.ac.uk/PROSPERO/display_record.php? RecordID = 76262).

### Selection criteria

All studies published in a peer‐reviewed journal with experimental designs were considered; thus, studies that did not provide information on a study design or were neither quantitative nor narrative in nature were not included. The type of experimental designs used included A‐B designs, randomised controlled trials and multiple baseline designs. Studies were selected if they attempted to formally evaluate the change in sleep following sleep interventions in adults aged 18 and over with a diagnosed ID. We included experimental studies, case series designs and case reports. ID had to be identified using local or international diagnostic criteria and terminology. Studies were required to assess sleep difficulties in adults with an ID although no restrictions were placed on the assessments used to evaluate sleep difficulties.

In studies that involved both children and adults, information on sleep for the adult participants had to be separately reported in the text or a table/figure (for at least one participant). Articles with a reported change in sleep noted as a secondary effect were excluded such as mental health problems (i.e. mood disorder, anxiety disorder, psychotic disorder, amnestic disorder, attention‐deficit hyperactivity disorder or personality disorder; Schutte‐Rodin, Broch, Buysse, Dorsey & Sateia 2008).

### Selection process

**Figure 1:**
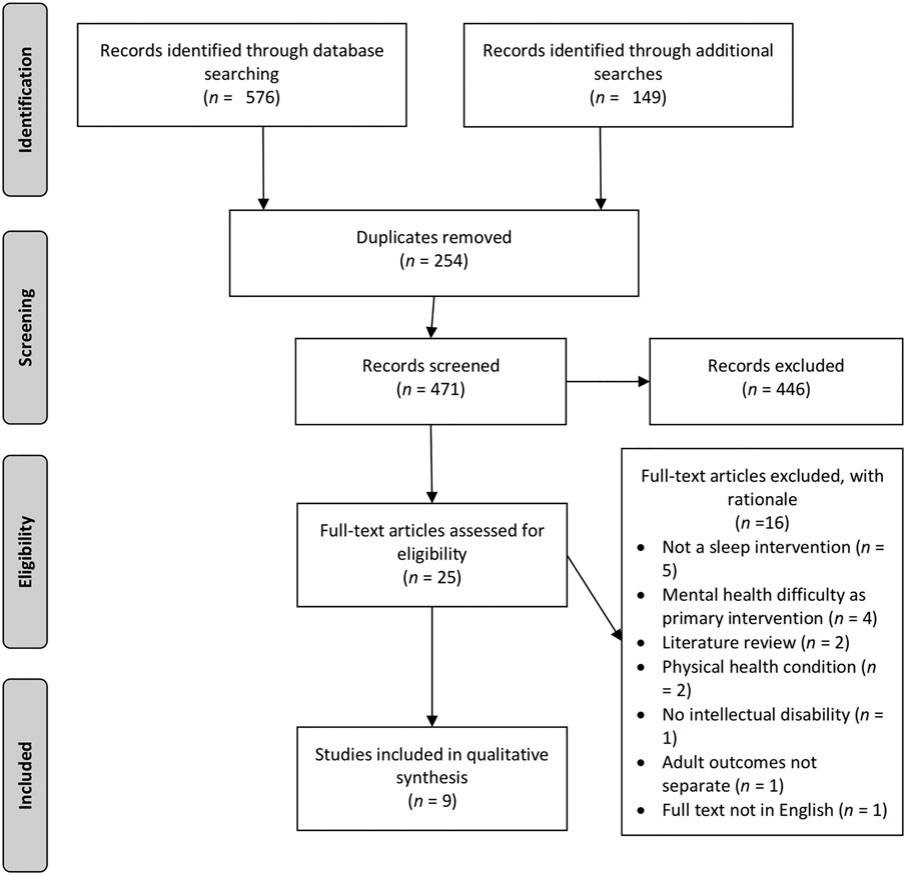
Preferred Reporting Items for Systematic Reviews and Meta‐Analyses flow chart of selection.

Two reviewers (P. S. and N. M.) independently screened the articles for eligibility (Figure 1). Disagreements in identified articles were resolved through consensus or failing this with the help of another reviewer (S. P.).

Articles that included children and adults without reporting adult data separately were contacted via email (*n* = 3). Those who subsequently provided adult‐only data were included in the review (*n* = 2).

### Data extraction and management

The following data were collated by the first researcher (P. S.) of the agreed journal articles for inclusion (Table 2):

- Participant characteristics: number of participants, study population (country, age, gender)
- Study characteristics: study design
- Intellectual disability criteria
- Sleep difficulty criteria
- Interventions used in the study
- Data on the efficacy of intervention

**Table 2:** Study characteristics

Authors	Sample				Study design	ID† criteria	Sleep difficulty criteria	Intervention type	Efficacy of intervention
	*N*	Country	Age	Gender (% male)					
Braam, Didden, Smits, and Curfs (2008)	51 (21 adults)	Netherlands	20–78 (adult only)	66.7	Randomised, placebo‐controlled study	Level of ID based on the age equivalent on the Social Functioning Scale for the Mentally Retarded	Sleep onset latency of 30 min or more, two or more periods of night waking's that lasted 45 min each night, five or more night waking's lasting more than 15 min each, during at least five nights each week. Present for the duration of at least 1 year	Melatonin (5 mg) or placebo	Significant change in sleep onset time (*P* < 0.01), decrease in sleep onset latency (*P* < 0.01), decrease in number of night wakes per night (*P* < 0.05), duration in night wakes (*P* < 0.05) and increase in total sleep time (*P* < 0.05)
Didden, Curfs, van Driel, and de Moor (2002)	4 (1 adult)	Netherlands	25 (adult only)	75	Case series (multiple baseline design)	Not given	‘Wake up several times’	Functional assessment and recommendations (extinction)	Visual analysis of data and reported improvement in night time disruptive behaviour
Gunning and Espie (2003)	155 (start) – >9 (completed)	UK	20–47	45.8	Case series (multiple baseline design)	From medical history	Structured assessment to diagnose sleep problems within framework of ICSD‐R‡	Optimal scheduling, sleep hygiene, stimulus control, relaxation, light therapy and cognitive behaviour therapy	Improvements to target variables found for six participants (*P* < 0.05)
Hare, Dodd, and Arshad (2008)	3	UK	43–69	66.6	Case series (AB design)	The Casemix Scale	Difficulty falling asleep, broken sleep, waking up during the night without any reason, waking in the early hours of the morning, reversal of sleep pattern, sleeping during the day and awake at night	Melatonin (6–12 mg§)	Improvement reported in sleep duration, however, not clinically significant
Hylkema and Vlaskamp (2009)	41 (34 adults)	Netherlands	19–66 (adult only)	63.4	Case series (AB design)	Level of ID categorised into moderate to severe (unclear diagnostic criteria)	Registration form with why it was thought had a sleep difficulty, sleep hygiene checklist, survey of activities, sleep diary and actigraphy	MDT¶ decided intervention, varying type: activity, different daily routines, sleep scheduling and other	Improvement in sleep efficiency and latency (*P* < 0.001)
Richings and Feroz‐Nainar (2010)	3	UK	32–74	33.3	Case series (series of individual case studies)	Not given	In text, not written separately: ‘poor sleep’, ‘sleeping poorly’, ‘difficulty falling asleep’	Melatonin (2.5–10 mg)	Increased agitation reported following use of melatonin
Short and Carpenter (1998)	1	UK	34	100	Case study	Not given	‘Difficulty getting off to sleep and waking frequently during the early hours of the morning, excessive daytime drowsiness and lethargy’	Light therapy	Sleep improvement reported
Stenfert‐Kroese and Thomas (2006)	2	UK	18–24	0	Case series (AB design)	Full Scale IQ††	Verbally reported. No formal assessment	Imagery rehearsal therapy	Participant verbally reported decrease in nightmares
Ward, Nanjappa, Hinder, and Roy (2015)	109 (23 adults, 2 adults without ID)	UK	18–67	65	Retrospective case note analysis	ICD‐10 (categorised as mild, moderate, severe or profound)	Type reported: ‘generally disturbed sleep, initial insomnia, early morning awakening, both initial insomnia and early morning awakening, reversed sleep pattern, awake all night, indication not recorded’	Melatonin (2.5–10 mg)	Sleep improvement reported in 15 of 23 participants

No *P* values are shown when not presented in paper.

† Intellectual disability.

‡ International Classification of Sleep Disorders – Revised.

§ Milligrams.

¶ Multi‐disciplinary team.

†† Intelligence quotient.

### Quality assessment

Quality assessment was completed by three researchers separately (P. S., N. M. and S. P.). The quality of each study and its most relevant study result were assessed using the Risk Of Bias In Non‐randomised Studies – of Interventions tool (ROBINS‐I; Sterne *et al*. 2016). The ROBINS‐I is a framework for assessing the risk of bias that may arise from confounding by selection of participants into the study, measurement of interventions, departures from intended interventions, missing data, measurement of outcomes and selection of reported results. Each domain is determined as having low, moderate, serious or critical risk of bias. The overall risk of bias was judged as the most severe risk of bias found in any domain. The exact interrater agreement between researchers of the first quality assessment completion was 95%. Reporting of the systematic review followed Preferred Reporting Items for Systematic Reviews and Meta‐Analyses (Moher *et al*. 2009) guidelines.

## Results

The initial search identified 725 articles. Once articles had been screened, nine were included in the systematic review. The main reasons for excluding articles following abstract review included the study not containing an intervention and/or having participants under 18. Figure 1 represents the Preferred Reporting Items for Systematic Reviews and Meta‐Analyses flow chart of the study selection. Table 2 is a summary of the study design, measurement of sleep difficulties used and interventions in the identified studies. Sixteen studies were excluded following a full text review. Reasons for exclusion following full text review included no sleep intervention (*N* = 5), mental health difficulty as the primary intervention (*N* = 4), literature review (*N* = 2), physical health condition (*N* = 2), no ID (*N* = 1), adult outcomes not separated from younger participants even after contacting the authors (*N* = 1) and article not published in English (*N* = 1).

Of the nine articles included, risk of bias was evaluated. Levels of risk across the six domains are summarised in Table 3. The overall risk of bias identified one study that had a low risk (Braam *et al*. 2008), two with serious risk (Short & Carpenter 1998; Gunning & Espie 2003) and six with critical risk of bias (Didden *et al*. 2002; Stenfert‐Kroese & Thomas 2006; Hare *et al*. 2008; Hylkema & Vlaskamp 2009; Richings & Feroz‐Nainar 2010; Ward *et al*. 2015).

**Table 3:** ROBINS‐I risk of bias assessment (low, moderate, serious, critical, no info)

Authors	Confounding	Selection	Measurement of intervention	Missing data	Measurement of outcomes	Reported result	Overall risk of bias
Braam *et al*. (2008)	Low	Low	Low	Low	Low	Low	Low
Didden *et al*. (2002)	Critical	Serious	Serious	Critical	Serious	Low	Critical
Gunning and Espie (2003)	Moderate	Low	Serious	Serious	Serious	Low	Serious
Hare *et al*. (2008)	Critical	Moderate	Serious	Low	Serious	No Info	Critical
Hylkema and Vlaskamp (2009)	Critical	Serious	Critical	Moderate	Serious	Moderate	Critical
Richings and Feroz‐Nainar (2010)	Critical	Low	Serious	Critical	No Info	Critical	Critical
Short and Carpenter (1998)	Serious	Low	Serious	No Info	No Info	No Info	Serious
Stenfert‐Kroese and Thomas (2006)	Critical	No Info	Serious	Serious	Critical	Serious	Critical
Ward *et al*. (2015)	Critical	Critical	Critical	Moderate	Critical	Serious	Critical

ROBINS‐I, Risk Of Bias In Non‐randomised Studies – of Interventions.

### Study characteristics

The number of adult participants in nine sleep studies varied from 1 to 34 (mean = 10). The total number of adult participants across studies was 97. The age range of the participants included was from 18 to 78 years (mean = 41 years). Severity of ID across all studies included mild (*N* = 6), moderate (*N* = 17), severe (*N* = 34), profound (*N* = 16), IQ 55–69 (*N* = 2) and not reported (*N* = 22). Of the 22 not reported, causes of ID included Down syndrome (*N* = 4), congenital rubella (*N* = 2), trauma (*N* = 1), Bardet–Biedl syndrome (*N* = 1), encephalitis 9 MND (*N* = 2), Prader–Willi syndrome (*N* = 1), hypothyroidism (*N* = 1) and perinatal problems (*N* = 1) and unknown (*N* = 9). Six studies were completed in the UK and three in the Netherlands.

Experimental designs included a randomised, placebo‐controlled study (*N* = 1; Braam *et al*. 2008), case series (*N* = 6; Short & Carpenter 1998; Gunning & Espie 2003; Stenfert‐Kroese & Thomas 2006; Hare *et al*. 2008; Hylkema & Vlaskamp 2009; Richings & Feroz‐Nainar 2010), case study (*N* = 1; Didden *et al*. 2002) and retrospective case note analysis (*N* = 1; Ward *et al*. 2015). Other medications (including aripiprazole, citalopram, carbamazepine, sodium valproate, diazepam, lamotrigine and cisapride) that participants were taking was reported in nine participants out of 97 (Short & Carpenter 1998; Hare *et al*. 2008; Richings & Feroz‐Nainar 2010). Nineteen participants were reported to have epilepsy, 19 participants diagnosed with autism and one had a diagnosis of attention‐deficit hyperactivity disorder. Visual impairment was reported in eight participants across all studies despite sampling not purposefully identifying this cohort.

### Identification of a sleep difficulty

One study assessed the type of sleep difficulty using a questionnaire created by the author based on the International Classification of Sleep Disorders Revised (Gunning & Espie 2003). One study used a combination of factors including a sleep diary, actigraphy, screening questionnaire and activity checklist (Hylkema & Vlaskamp 2009). Braam *et al*. (2008) utilised a sleep diary completed by family or carers to assess the sleep difficulty measuring sleep onset latency, sleep onset time, wake time, total sleep time, number and duration of night wakes. Five studies used verbal report of the individuals being assessed or their carers including sleep onset latency, night waking or subjective quality such as *poor sleep* (Short & Carpenter 1998; Didden *et al*. 2002; Stenfert‐Kroese & Thomas 2006; Hare *et al*. 2008; Richings & Feroz‐Nainar 2010; Ward *et al*. 2015).

### Measurement of sleep difficulties

A Cambridge Neurotechnology Actiwatch was used to measure duration sleeping, duration awake, sleep efficiency, number of sleep bouts, number of wake bouts, fragmentation index and hours of sleep onset latency in two studies (Hare *et al*. 2008; Hylkema & Vlaskamp 2009). Didden *et al*. (2002) reported number of minutes of behaviours associated with night‐time disruption caused using an individualised monitoring form. Gunning and Espie (2003) reported different measurement types across all participants in the study including sleep onset latency, night wakes, total sleep time, caffeine consumption and number of episodes of night bedclothes removal using individualised sleep diaries completed by carers. Other measures used included carer sleep quality rating and morning sleepiness rating (Gunning & Espie 2003). Richings and Feroz‐Nainar (2010) relied on the difficulties being verbally reported to clinicians. Short and Carpenter (1998) did not specify how sleep difficulties were measured. Stenfert‐Kroese and Thomas (2006) utilised participant self‐report for frequency of nightmares per week. Braam *et al*. (2008) used a diary completed by a patient or carer focusing on sleep onset latency, number of night wakes, sleep onset time, wake up time, number and duration of night wakes and total sleep time. Ward *et al*. (2015) utilised carer verbal reports and in some cases a sleep diary.

Three studies assessed the differences between baseline and follow up statistically (Gunning & Espie 2003; Braam *et al*. 2008; Hylkema & Vlaskamp 2009), two created categories according to whether they felt participants' sleep had improved (Didden *et al*. 2002; Hare *et al*. 2008) and four assessed differences qualitatively (Short & Carpenter 1998; Stenfert‐Kroese & Thomas 2006; Richings & Feroz‐Nainar 2010; Ward *et al*. 2015).

**Figure 2:**
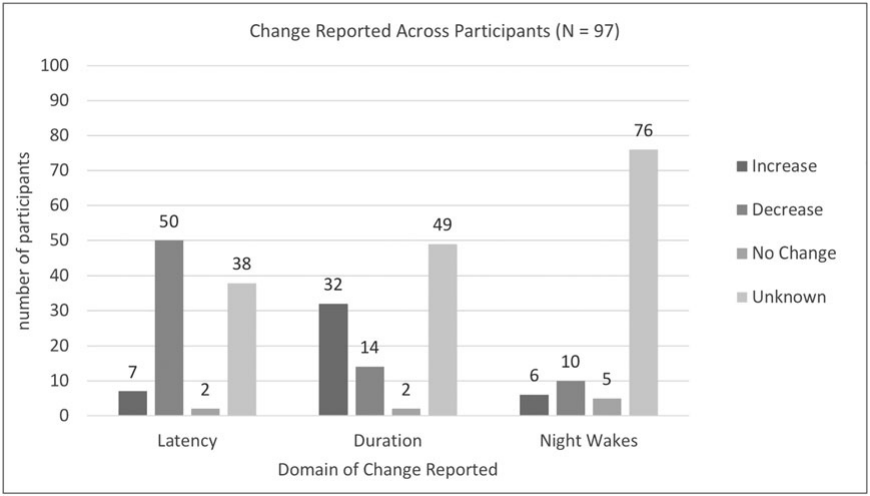
Reported change in key domains.

As defined by the International Classification for Diseases – 10th Revision criteria for a sleep disorder, the amount, quality or timing of sleep is required to be affected (World Health Organisation 1992). Figure 2 summarises the number of participants across nine sleep intervention studies in which the sleep onset latency, duration and number of night wakes were recorded prior and after intervention. Sleep onset latency was only recorded in 58 participants, sleep duration was measured in 49 participants and number of night wakes was reported in 16 participants across all included studies.

### Interventions for sleep difficulties

Across the nine studies, interventions were not directly comparable. Either interventions varied across studies or the outcome measures used were different. The following is a summary of the main intervention methodologies evaluated in the included articles.

- Melatonin

Melatonin was used in four studies and a total of 40 participants in varying doses (2.5–12 mg; Braam *et al*. 2008; Hare *et al*. 2008; Richings & Feroz‐Nainar 2010; Ward *et al*. 2015). Braam *et al*. (2008), which had a low risk of bias, identified that in eleven adult participants, 5 mg of melatonin was significantly (difference in change 29.48; *P* < 0.01) more effective than a placebo (*N* = 10) at decreasing sleep onset latency. Ward *et al*. (2015) had a serious risk of bias in the reported result, which identified ‘mild’, ‘good’ and ‘excellent’ improvements in 15 of the 23 adult participants who were prescribed between 2.5 and 10 mg of melatonin. In one case series with ‘critical’ risk of bias, all three participants stopped taking melatonin due to agitation (Richings & Feroz‐Nainar 2010). The final case series with three participants did not report enough information to make an informed decision about risk of bias (Hare *et al*. 2008). Improvements in sleep duration were noted in two of the three participants; however, conclusions about overall efficacy could not be established (Hare *et al*. 2008).

- Functional assessment

One study identified completed a functional assessment (through interviews and behaviour recording) and implemented an extinction intervention one 25‐year‐old (Didden *et al*. 2002). Extinction involves removing a reinforcer that maintains a behaviour (Didden *et al*. 2002). This found a decrease in ‘night time disruption’; however, it was unclear if this increased sleep quality across other domains such as latency or duration (Didden *et al*. 2002).

- Mixed methods

Two studies used multiple or specific methods depending on the presenting sleep difficulty (Gunning & Espie 2003; Hylkema & Vlaskamp 2009). The intervention components utilised with nine participants identified by Gunning and Espie (2003) were ‘optimal scheduling’, ‘sleep hygiene’, ‘stimulus control’, ‘relaxation’, ‘light therapy’ and ‘cognitive behaviour therapy’. Six of the nine intervention packages produced a statistically significant effect on one of the outcome measures evaluated with varying effect sizes and levels of significance (Gunning & Espie 2003). Hylkema and Vlaskamp (2009) utilised methods agreed on by a multi‐disciplinary team including varying activities, different daily routines, sleep scheduling and other with 34 participants. Statistically significant (*P* < 0.001) improvements were noted in sleep efficiency and latency (Hylkema and Vlaskamp 2009).

- Other

Light therapy was used in one case study (Short & Carpenter 1998). Risk of bias was unable to be established due to lack of information. The authors reported that following 2 weeks, sleep patterns were reported to be normal and remained stable (Short & Carpenter 1998). Imagery rehearsal is the final intervention used in a case series with two participants (Stenfert‐Kroese & Thomas 2006). The participants reported no further nightmares following 2 weeks of intervention and at 6 months (Stenfert‐Kroese & Thomas 2006).

## Discussion

### Primary findings

This systematic review aimed to identify if sleep interventions in adults with an ID improved sleep. Across all reviewed studies, seven reported that sleep interventions improved the sleep of their participants. However, there was a notable heterogeneity between studies in terms of sleep assessments, population studied, sleep intervention utilised, outcome reported, medications reported and analysis of results. This heterogeneity makes it difficult to synthesise a clear message about which sleep interventions work well for adults with an ID. Furthermore, findings from this study also indicated that the overall quality of studies in this field is low. Of the included studies, only one was evaluated to have an overall low risk of bias. The remaining studies had a serious to critical risk of bias.

It is not surprising that there is a perception that sleep difficulties in this population cannot be changed (O'Reilly 1995; Wiggs & Stores 1996; Symons, Davis & Thompson 2000). Adding to the complications, the methods identifying sleep difficulties varied significantly. Actigraphy has been correlated with polysomnography and indicated as an objective measure of sleep in both drug and non‐pharmacological interventions (Sadeh 2011; Mantua, Gravel & Spencer 2016; Riemann *et al*. 2017). Despite the weight of evidence and a population who often cannot verbally report their experience, only two studies with 37 adults utilised actiwatches (Hare *et al*. 2008; Hylkema & Vlaskamp 2009; Martin & Hakim 2011).

### Sleep hygiene and behavioural interventions

Despite several published randomised trials of behavioural interventions with children, no high‐quality studies have assessed behavioural interventions in adult populations with an ID (Wiggs & Stores 1998; Montgomery, Stores & Wiggs 2004).

Current interventions that authors have indicated efficacy amongst some sleep parameters and participants in the current literature include optimal night time or sleep scheduling, different daily routines, varying activities, sleep hygiene, stimulus control, relaxation, light therapy and cognitive behaviour therapy (Gunning & Espie 2003; Hylkema & Vlaskamp 2009). Gunning and Espie (2003) specified criteria for the use of specific interventions. Hylkema and Vlaskamp (2009) relied on an MDT decision‐making process, leading to a lack of clarity about which interventions are indicated or effective for specific sleep difficulties. Despite being recommended, no functional analysis procedure has been identified in the current review for behaviours that challenge that impact on sleep or as part of a sleep intervention study (Wiggs & Stores 1998; Iwata & Dozier 2008; National Institute for Health and Care Excellence 2015). However, functional assessments have shown an initial benefit, but more studies investigating this with a variety of functions for behaviour are necessary (Didden *et al*. 2002).

### Melatonin

Melatonin was the only medication evaluated in the current review for sleep difficulties in adults with an ID. Although the highest quality evidence indicates benefits in sleep onset latency for the use of melatonin in the population, potential agitation, aggression and restless were noted in a small number of cases (Braam *et al*. 2008; Hare *et al*. 2008; Richings & Feroz‐Nainar 2010; Ward *et al*. 2015). Similar mixed results in evidence have been found by Braam *et al*. (2009) in a meta‐analysis for the use of melatonin. These mixed results could be attributable to individual differences in receptor sensitivity as a causative factor amongst adults with an ID (Pandi‐Perumal *et al*. 2008).

### Future research and clinical implications

The goal of this study was to examine the effectiveness of sleep interventions on sleep in adults with an ID. There is evidence, however, that sleep improvement can be a secondary consequence of mental health or lifestyle interventions (Altabet, Newman & Watson‐Johnson 2001; Biswas, Bhaumik & Branford 2001; Verhoeven, Egger, Willemsen, de Leijer & Kleefstra 2012; Wilhite, Biren & Spencer 2012; Majeske, Garakani, Maloutas, Bryson & Kellner 2013). As having an ID is frequently co‐morbid with other physical, behavioural and mental health conditions, sleep disruption is often an indirect consequence of co‐morbidity. For instance, results from studies focusing on fitness, depression and bipolar disorder indicate positive effects on sleep following interventions (Altabet *et al*. 2001; Wilhite *et al*. 2012; Majeske *et al*. 2013; Hamers, Festen & Hermans 2018). This indicates that the improvement of sleep in adults with an ID may involve a careful consideration of co‐occurring morbidities and what works for those morbidities. While investigating sleep improvement as an indirect improvement of other interventions was beyond the scope of this review, a broader review on interventions and sleep improvement in adults with ID could represent an interesting avenue for future research. In addition, including individual with co‐morbid mental health conditions and of course children with ID would also provide more information regarding the best interventions for sleep disturbances.

The use of melatonin and other medications that are commonly prescribed lead to polypharmacy in practice for sleep difficulties that may make evaluating individual components more difficult (Lake, Balogh & Lunsky 2012; National Institute for Health and Care Excellence 2013; Ward *et al*. 2015). In the UK, in line with stopping over medicating people with learning disability (https://www.england.nhs.uk/learning-disabilities/improving-health/stomp/), standardised pathways and medicating practices should be considered for treating sleep along with mental health conditions. Reducing variations in prescribing practices across a larger sample should be considered to strengthen the evidence for medication in sleep difficulties.

As heterogeneous data collected throughout studies have hindered a clear synthesis of efficacy, the use of a standardised ID sleep diary or actigraphy should be used in clinical practice and research (Riemann *et al*. 2017). There is also a need to compare medications, such as melatonin, and function‐based behavioural interventions to ‘treatment as usual’ for sleep difficulties in adults with ID (Asaria, Griffin & Cookson 2016). This should also take into account differences in presenting problems including physical and mental health concerns (Benca 2005; Matson *et al*. 2007; Walker 2009; Freeman *et al*. 2017).

### Strengths and limitations

The strengths of this study are that it is the first to systematically assess sleep interventions for adults with an ID. The present study searched a wide variety of electronic databases in the search criteria with a large variety of search terms. Unfortunately, the ability to draw robust conclusions about the potential efficacy of interventions are significantly hindered by the heterogeneity of data and low quality of included studies. The use of a randomised study while using the ROBINS‐I is also a potential limitation as it may underestimate the risk of bias of that particular study. This may indicate that there is a stronger evidence base for the use of melatonin that may not always be indicated. There is also a risk of publication and language biases for current literature.

## Conclusions

The evidence for assessing the effectiveness of sleep interventions for adults with an ID has significant heterogeneity and was generally of poor quality. Because of the paucity of evidence, recommendations when adults with an ID present with a sleep difficulty or sleep disorder relies on clinicians' anecdotal evidence. However, there is an opportunity to research standardised interventions using actigraphy and a standardised sleep diary in order to address heterogeneity. In addition, medication rationalisation, epilepsy optimisation and regular health checks should be undertaken thoroughly to manage additional causes of poor sleep. Clinicians should also, where possible, screen for sleep difficulties following referrals for behaviours that challenge or mental health disorders when not responding to typical interventions.

## Conflict of Interest

The authors declare no conflicts of interest in publishing this work.
